# Associations of serum uric acid level and gout with cardiac structure, function and sex differences from large scale asymptomatic Asians

**DOI:** 10.1371/journal.pone.0236173

**Published:** 2020-07-20

**Authors:** Kuo-Tzu Sung, Chi-In Lo, Yau-Huei Lai, Jui-Peng Tsai, Chun-Ho Yun, Chih-Chung Hsiao, Jen-Yuan Kuo, Charles Jia-Yin Hou, Ta-Chuan Hung, Cheng-Huang Su, Chung-Lieh Hung, Hung-I Yeh

**Affiliations:** 1 Division of Cardiology, Department of Internal Medicine, MacKay Memorial Hospital, Taipei, Taiwan; 2 Department of Medicine, Mackay Medical College, New Taipei City, Taiwan; 3 Institute of Clinical Medicine, National Yang-Ming University, Taipei, Taiwan; 4 Mackay Medicine, Nursing, and Management College, Taipei, Taiwan; 5 Division of Cardiology, Department of Internal Medicine, MacKay Memorial Hospital, Hsinchu, Taiwan; 6 Division of Radiology, MacKay Memorial Hospital, Taipei, Taiwan; University of Dundee, UNITED KINGDOM

## Abstract

Hyperuricemia (HU) is a marker for heart failure. There are relatively few data in the Asian population regarding the effects of hyperuricemia and gouty disorders on cardiac remodeling and diastolic dysfunction (DD), an intermediate stage in the development of heart failure. We consecutively recruited asymptomatic Asian individuals to undergo cardiovascular surveys. We categorized them into Non-HU, HU, and Gout groups. We measured cardiac structure and indices for diastolic function, including tissue Doppler (TDI)-derived LV e’ and E/e’. Among 5525 participants, 1568 had HU and 347 had gout. The presence of gout and higher uric acid levels (SUA) (<4, 4–6, 6–8, 8–10, > = 10 mg/dL) were associated with greater LV wall thickness, greater LV mass/volumes, larger LA volume, lower LV e’ and higher E/e’. Higher SUA was associated with greater LV mass index (adjusted coefficient: 0.37), greater mass/volume ratio (adjusted coefficient: 0.01) and larger LA volume index (adjusted coefficient: 0.39, all p<0.05). Both HU and Gout groups were associated with lower LV e’ (coefficient: -0.086, -0.05), higher E/e’ (coefficient: 0.075, 0.35, all p <0.05), larger LA volume, and higher DD risk (adjusted ORs: 1.21 and 1.91 using Non-HU as reference, respectively, both p <0.05). SUA set at 7.0 mg/dL provided the optimal cut-off for identifying DD, with markedly lower e’ (HU: 8.94 vs 8.07, Gout: 7.94 vs 7.26 cm/sec) and higher LV E/e’ in HU/Gout women than in men (HU: 7.84 vs 9.79 cm/sec for men and women, respectively, all p <0.05). Hyperuricemia, even at a relatively low clinical cut-off, was associated with unfavorable remodeling and was tightly linked to diastolic dysfunction. The presence of gout likely aggravated these conditions. Women with hyperuricemia or gout had worse diastolic indices than men despite similar degrees of LV remodeling.

## Introduction

Circulating serum uric acid (SUA) is the final product of purine metabolism and is closely associated with a variety of metabolic abnormalities and coronary artery disease [1, 2]. Several lines of evidence suggest that increased SUA levels may be observed in heart failure (HF) and may be tightly associated with higher incidence of adverse cardiovascular events [3–6]. Studies reported the independent role of circulating SUA levels on LV remodeling or hypertrophy, likely mediated by several pathological mechanisms, including stimulation of inflammatory responses, activation of the renin-angiotensin system (RAS), production of oxidative stress, and suppression of NO release [7, 8].

Left ventricular remodeling and hypertrophy are thought to be adverse cardiac adaptations in response to stress, pressure-, or volume-overload conditions [9]. Unfavorable LV remodeling and hypertrophy are associated with chamber stiffness with resultant myocardial relaxation abnormalities and diastolic dysfunction (DD) [9], a potential precursor of HF [10]. While hyperuricemia may act as mediator of gouty disorder, the presence of gout rather than hyperuricemia *per se* was proposed to be more directly linked to DD [11]. Nevertheless, no studies have examined these associations in a large-scale ethnic Asian population. Therefore, we performed the present prospective study.

## Materials and methods

### Study population

We studied consecutive patients undergoing annual cardiovascular surveys and health evaluations (from June 2009 to March 2012) at MacKay Memorial Hospital, Taipei, a tertiary medical center in Northern Taiwan. All study participants underwent comprehensive physical exams, chest radiography, 12-lead ECG, biochemical analysis and transthoracic echocardiography with tissue Doppler imaging (TDI). Baseline clinical characteristics and all biochemical data, including uric acid level, and echocardiography were examined on the same day. Structured questionnaires were administered to obtain information on subjective symptoms, lifestyles and detailed past medical histories. All the participants provided written informed consent. We excluded subjects with significant valvular heart disease, pulmonary hypertension (systolic pulmonary arterial pressure, >50 mmHg), histories of prior cardiac surgery, congenital heart disease, permanent pacemaker implantation, LV ventricular ejection fraction (LVEF) <50% or clinical diagnosis of clinical heart failure (HF), and those receiving regular hemodialysis. The investigation conformed to the principles outlined in the Declaration of Helsinki and the local ethics committee (MacKay Memorial Hospital) approved the study (number: 14MMHIS202). The descriptions of our study settings were further detailed in a previous publication [12]. Hypertension (HTN) was defined as the presence of known history of hypertension from questionnaire by self-report, or confirmed clinical diagnosis with systolic blood pressure (SBP) ≥140mmHg or diastolic blood pressure (DBP) ≥90mmHg on two occasions, or those with active pharmaceutical control for HTN. Presence of diabetes mellitus (DM) was defined as known DM history from questionnaire or previous diagnosis of DM with ongoing pharmaceutical control. Hyperlipidemia treatment was defined as ongoing use of anti-lipid drugs, ie. statins or fenofibrates.

#### Definitions of hyperuricemia and gout

We further categorized our study cohort into three groups based on circulating SUA levels and medical history of gout: 1) Non-hyperuricemic Group (Non-HU): serum uric acid (SUA) level ≤6 mg/dL in women and ≤7 mg/dL in men without any known prior gout history; 2) Hyperuricemic Group (HU): SUA >6 mg/dL in women and >7 mg/dL in men without known gout history; and 3) Gout: individuals with known gouty arthritis or current medication use for gouty disorder. The clinical diagnosis of gout was based on ACR guidelines: at least one episode of peripheral joint or bursal swelling, pain, or tenderness with either presence of tophus or monosodium urate monohydrate crystals in symptomatic joint/bursa or a threshold score ≥8 in different domains in the new American College of Rheumatology/European League Against Rheumatism (ACR/EULAR) gout classification criteria [13].

#### Conventional echocardiography and diastolic functional assessment

All echocardiographic studies were acquired using commercially available ultrasound Hewlett-Packard instruments (Sonos 5500, Philips, The Netherlands) or GE systems (Vivid i) equipped with 2–4-MHz transducers (3S-RS) during the recruitment period by a single experienced technician blinded to clinical information. All echocardiographic measures were obtained through consecutive three heart cycles. Cardiac chamber dimensions, LV and left atrial (LA) volumes (LAV), LVEF as well as LV mass (LVM) were measured according to current guidelines of the American Society of Echocardiography [14]. Relative wall thickness (RWT) was calculated as ratio of two-times posterior wall thickness at end-diastole divided by LV end-diastolic diameter using M-mode from parasternal long axis view and expressed as percentage. LV mass index (LVMi) (g/m^2^) and indexed LA volume (LAVi) were defined as the ratio of LV mass and LAV to body surface area, respectively. LV hypertrophy was defined as LVMi >95 mg/m^2^ in women and >115 mg/m^2^ in men.

We further classified the participants into normal, concentric remodeling, and left ventricular hypertrophy groups based on RWT and LV mass index. Normal group: RWT ≤0.42 and no LVH; Concentric remodeling: RWT >0.42 and no LVH; LV hypertrophy: individuals with LVH. Diastolic functional evaluation included pulsed wave Doppler sampling positioned at the mitral leaflet tip area (mitral inflow area) with early (E), and the late diastolic (A) filling velocities ratio (E/A ratio). Tissue Doppler imaging (TDI)-based mitral annular relaxation velocity (e’) of ventricular septal and lateral segments were also obtained. Grading of diastolic dysfunction was then determined according to European Association of Echocardiography/American Society of Echocardiography (EAE/ASE) recommendations. We further graded diastolic function as follows: Normal: septal e’ ≥8 cm/s and lateral e’ ≥10 cm/s and E/e’ ≤8; mild diastolic dysfunction: septal e’ <8 cm/s or lateral e’ <10 cm/s and deceleration time (DT) >200 ms and E/e’ ≤8; moderate-to-severe diastolic dysfunction: septal e’<8 cm/s or lateral e’<10 cm/s and DT ≤200 ms and E/e’ >8 [13].

### Statistical analysis

**Fig 1:**
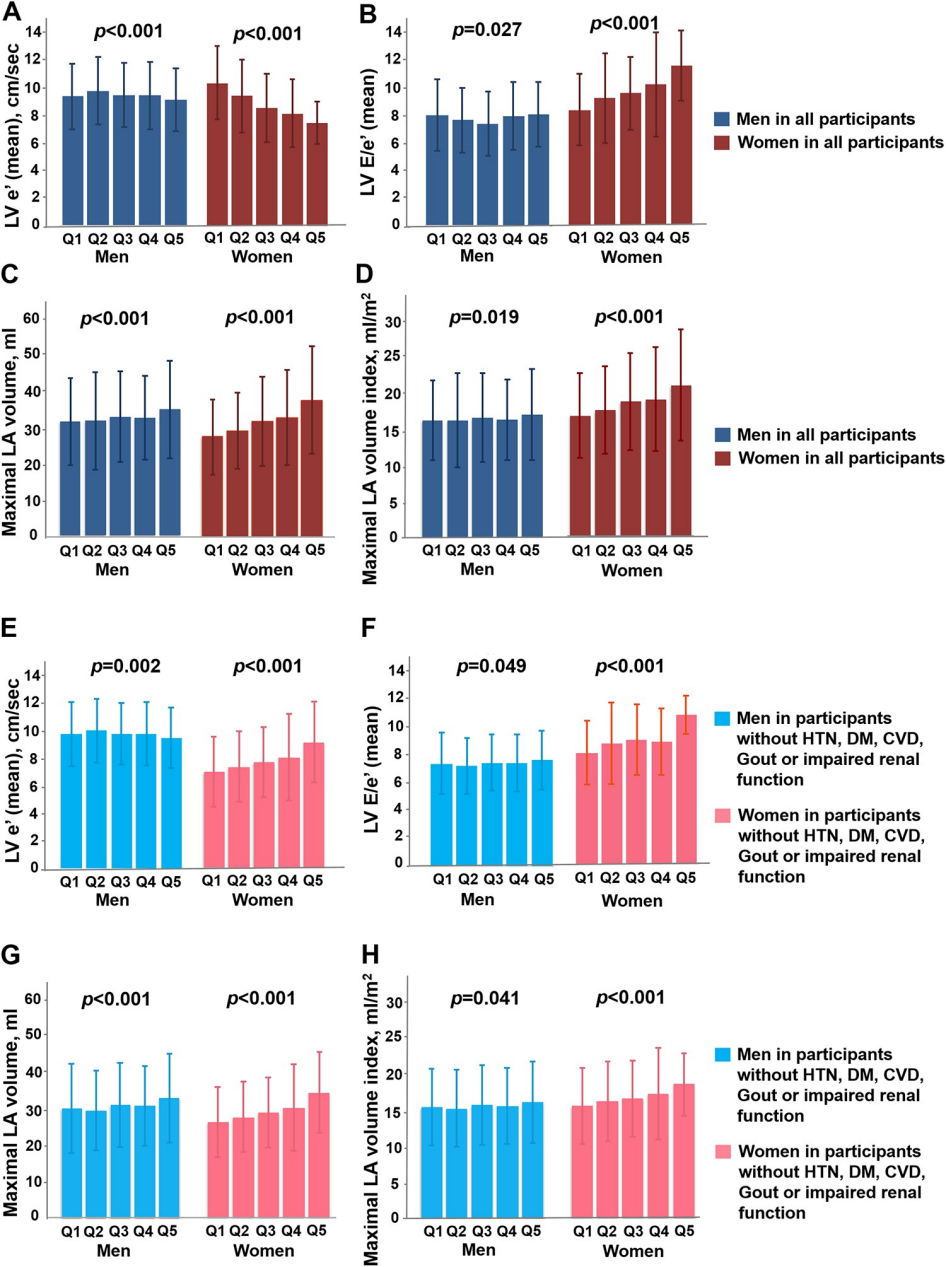
Associations among serum uric acid level, cardiac structural remodeling and diastolic functional indices. Lower mitral annular relaxation e’, higher LV E/e’ and greater LA volume (with and without index) across higher uric acid quintiles in both genders for the whole cohort (n = 5525) (A-D) and a subset free from systemic or cardiovascular diseases (n = 3912) (E-H), with women demonstrating more pronounced changes of LV e’, LV E/e’ and LA volume across uric acid quintiles.

Data were expressed as mean ± SDs for continuous variables and percentage (%) for categorical variables. The clinical characteristics across Non-HU, HU and Gout categories (Table 1) and echocardiography-derived parameters based on ordered clinical SUA cut-off values (SUA level: <4, 4–6, 6–8, 8–10, >10 mg/dL, Table 2) or SUA quintiles (Fig 1A–1D) were tested using Cuzick’s nonparametric test. The chi-square test was used for comparisons of distributions for categorical variables across three study groups (arranged as Non-HU, HU, and Gout). We also examined the conventional echocardiography-derived measures and diastolic functional indices (presented as unadjusted and adjusted means, Table 2) using one-way ANOVA to compare baseline characteristics across three groups with paired comparisons made among groups using Bonferroni post-hoc corrections. For diastolic dysfunction (DD) and assessment of ventricular geometric remodeling (as dependent variables), we performed multivariate linear (coefficient values and 95% confidence interval [95% CI] reported) and logistic regression analyses (odds ratios [OR] and 95% confidence interval [95% CI] reported) after adjusting for baseline covariates (CV) including age, gender, body mass index, blood pressure, fasting glucose, HDL, total cholesterol, estimated glomerular filtration rate (eGFR), and histories of hypertension, diabetes, hyperlipidemia treatment, or coronary artery disease (as independent variables). These same clinical covariates (CV) were also used in adjusted means analysis.

**Table 1:** Basic information and echocardiographic measurement of cardiac structure and diastolic function for study participants in normal individuals, asymptomatic hyperuricemia, and those with gout.

	Non-HU	HU	Gout	*Trend p value*
Number, n	n = 3610	n = 1568	n = 347	
**Demographic and clinical characteristics**				
Age, years	48.94±11.12	50.65±12.25*	52.24±10.21*	<0.001
Female, number (%)	1467 (40.6%)	460 (28.0%)*	41 (17.9%)*#	<0.001
BMI, kg/m^2^	23.74±3.35	25.60±3.93*	26.08±3.86*	<0.001
SBP, mmHg	120.99±17.77	126.14±16.62*	127.56±19.42*	<0.001
DBP, mmHg	74.21±11.23	77.45±12.23*	78.96±12.87*	<0.001
Pulse pressure, mmHg	89.81±12.50	93.69±13.66*	95.16±14.25*	<0.001
Heart rate, bpm	66.28±11.27	68.46±19.15	67.15±11.55	<0.001
Fasting glucose, mg/dL	100.36±21.35	102.55±21.65*	105.10±27.67*	<0.001
Uric acid, mg/dL	5.27±1.03	7.51±0.99*	7.21±1.82*#	<0.001
Total cholesterol, mg/dL	198.66±35.57	209.34±39.12*	205.16±37.61*	<0.001
Triglyceride, mg/dL	122.97±81.37	161.94±151.97*	166.90±99.52*	<0.001
LDL, mg/dL	126.69±32.37	138.12±33.40*	134.33±34.74*	<0.001
HDL, mg/dL	55.90±15.47	47.99±12.09*	48.31±13.72*	<0.001
eGFR, mL/min/1.73m^2^	90.92±17.04	82.10±82.10*	81.06±17.44*	<0.001
WBC count, 10^3^/uL	6.01±1.59	6.57±1.59*	6.55±1.72*	<0.001
Hemoglobin, mg/dL	14.11±1.53	14.60±1.45	14.16±1.50	<0.001
Hs-CRP, ml/L	0.19±0.46	0.27±0.47*	0.26±0.59	<0.001
NT-ProBNP, ng/mL	47.19±101.01	48.53±165.26	53.94±124.07	0.417
HTN, number (%)	533 (14.8%)	464 (29.6%)*	124 (36.0%)*#	<0.001
DM, number (%)	201 (5.6%)	180 (11.5%)*	49 (14.4%)*#	<0.001
CVD, number (%)	191 (5.3%)	162 (10.3%)*	49 (14.4%)*#	<0.001
Hyperlipidemia treatment, number (%)	213 (5.9%)	211 (13.5%)*	82 (23.62%)*#	<0.001

Trend p <0.05means significant difference across three groups, 95% confident interval;

*: p<0.05 compared to normal group.

^**#**^: p<0.05 compared to hyperuricemia group, 95% confident interval (CI). BMI: body mass index; SBP: systolic blood pressure; DBP: diastolic blood pressure; LDL: low-density lipoprotein; HDL: high-density lipoprotein; GFR: estimated glomerular filtration rate; hs-CRP: high-sensitivity CRP; HTN: hypertension; DM: diabetes mellitus; CVD: cardiovascular disease.

**Table 2:** Linear regression models on the relationship among uric acid, cardiac structural remodeling and diastolic indices.

	UA Categories (mg/dL)						Univariate Regression		Multivariate Regression		Multivariate Regression^※^	
							(per 1mg/dL UA +)		(per 1mg/dL UA +)		(per 1mg/dL UA +)	
Mean (SD)	<4mg/dL	4-6mg/dL	6-8mg/dL	8-10mg/dL	>10mg/dL	p for trend	*Coef*.	p value	*Coef*.	p value	*Coef*.	p value
IVS, mm	8.5±1.1	8.9±1.1	9.3±1.0	9.5±1.4	9.6±1.2	<0.001	0.21	<0.001	0.03	0.017	0.02	0.047
LVEDV, mL	69.4±14.1	74.1±13.8	79.6±13.6	82.5±13.6	83.016.9	<0.001	2.75	<0.001	-0.076	0.617	-0.07	0.641
LVESV, mL	25.4±7.7	27.6±7.1	30.0±7.4	31.7±7.7	32.3±10.1	<0.001	1.26	<0.001	-0.011	0.902	-0.017	0.85
LVEF, %	67.8±4.7	67.4±4.5	67.0±4.7	66.2±5.0	65.4±5.9	<0.001	-0.26	<0.001	-0.028	0.651	-0.02	0.76
LV mass, gm	124.0±30.5	137.5±32.0	152.9±30.1	160.7±31.8	163.3±35.2	<0.001	7.61	<0.001	0.227	0.502	0.06	0.87
LV mass/volume, gm/ml	1.79±0.27	1.87±0.28	1.93±0.27	1.97±0.34	2.01±0.27	<0.001	0.04	<0.001	0.01	0.037	0.03	0.36
Relative wall thickness, %	38.0±4.6	38.8±4.6	39.2±4.5	39.5±5.3	40.3±4.4	<0.001	0.29	<0.001	0.12	0.044	0.16	0.01
LVMi, gm/m^2^ ¥	72.8±14.5	75.9±14.7	78.2±14.4	78.7±14.4	82.2±18.5	<0.001	1.25	<0.001	0.37	0.034	0.28	0.11
Peak E wave, cm/s	75.7±16.7	70.5±16.6	67.0±15.3	65.5±15.1	69.9±17.4	<0.001	-1.92	<0.001	-0.17	0.437	-0.16	0.46
Peak A wave, cm/s	58.8±16.2	61.0±18.7	60.6±20.3	61.4±19.1	63.1±19.4	<0.001	0.24	0.198	0.4	0.078	0.77	0.02
E/A ratio	1.37±0.46	1.25±0.45	1.19±0.40	1.14±0.38	1.15±0.55	<0.001	-0.04	<0.001	-0.04	<0.001	-0.01	0.002
Deceleration time, ms	201.2±35.2	204.1±39.2	204.2±39.3	206.4±40.2	203.8±57.1	<0.001	0.52	0.18	-0.19	0.71	-0.45	0.38
IVRT, ms	86.4±14.9	89.5±14.9	90.2±15.2	92.0±14.9	86.5±20.2	<0.001	0.81	<0.001	0.06	0.783	0.04	0.83
LV e’ (mean), sec	10.1±2.5	9.3±2.5	9.0±2.4	8.8±2.2	8.3±2.3	<0.001	-0.24	<0.001	-0.09	<0.001	-0.15	<0.001
LV E/e’ (mean)	8.0±2.4	8.1±2.7	8.1±2.6	8.4±2.5	9.0±2.6	<0.001	0.14	<0.001	0.075	0.019	0.13	<0.001
Maximal LAV, ml	27.88±10.75	29.84±11.94	31.95±12.28	31.88±11.58	34.22±13.27	<0.001	1.50	<0.001	0.16	0.21	0.14	0.30
Maximal LAVi, ml/m^2^ ¥	16.12±5.62	16.39±6.21	16.69±6.20	16.34±5.77	16.86±6.29	<0.001	0.22	<0.001	0.39	<0.001	0.37	<0.001

^**※**^ Sensitivity analysis after excluding hyperuricemia/gout medication use;

^¥^ BMI was not included in multi-variate models;

Multivariate models: adjusted for age, sex, body mass index, systolic blood pressure, heart rate, fasting blood glucose, estimated glomerular infiltration rate, low-density lipoprotein, high-density lipoprotein, diabetes, hypertension, hyperlipidemia treatment, cardiovascular disease.

LV: left ventricular; IVS: interventricular septum; LAV: left atrial volume; LAVi: indexed left atrial volume; LVEDV: left ventricular end-diastolic volume; LVESV: left ventricular end-systolic volume; LVMi: LV mass index; SV: stroke volume; LVEF: left ventricular ejection fraction; LAV: left atrial volume; DT: deceleration time; IVRT: Isovolumic relaxation time; E/e’: mitral inflow velocity E divided by E/e’ (mean).

Statistical analyses were performed using STATA statistical software (version 13; Stata Corp). Two-sided values of *p* <0.05 were considered statistically significant.

## Results

### Baseline characteristics

There were 5525 asymptomatic participants (mean aged 49.63 ± 11.44 years, 35.6% were women). The baseline characteristics of these three groups are displayed in Table 1. Subjects with hyperuricemia and gout tended to be older, have greater body mass index, higher blood pressure components (systolic, diastolic, and pulse pressure), higher fasting glucose levels, and worse lipid profiles (higher total cholesterol or LDL and lower HDL levels) (all p-values for trend <0.001). Participants in the HU and Gout groups also showed higher hs-CRP levels than those of the Non-HU group (0.19 ± 0.46 vs 0.27 ± 0.47 vs 0.26 ± 0.59 ml/L, for Non-HU, HU and Gout, respectively, p <0.001). Subjects in the HU and Gout groups had more co-morbidities, including hypertension, diabetes, dyslipidemia, and coronary artery disease (all p <0.001).

### Hyperuricemia or gout on cardiac geometry and diastolic dysfunction

**Fig 2:**
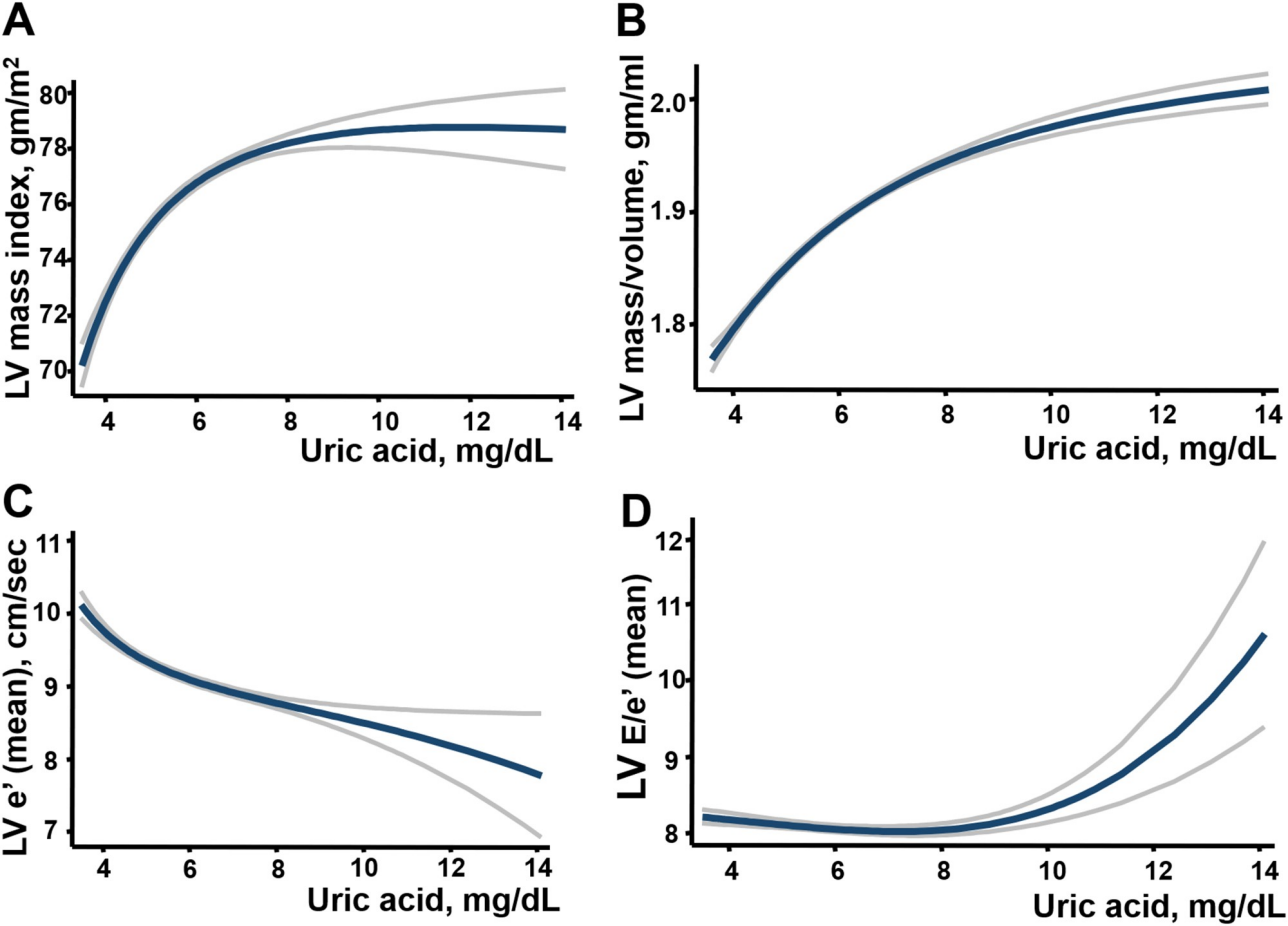
The associations among serum uric acid, echocardiographic measurement of LV diastolic function (DD) and remodeling. Models were adjusted for age, sex, body mass index, systolic blood pressure, heart rate, fasting blood glucose, estimated glomerular infiltration rate, total cholesterol, high-density lipoprotein, diabetes, hypertension, hyperlipidemia, and coronary artery disease (A-D). In LV mass index model, body mass index (BMI) was not included. The blue line represents the cubic spline with the gray lines represents the upper and lower limit of 95% confidence interval (CI).

**Fig 3:**
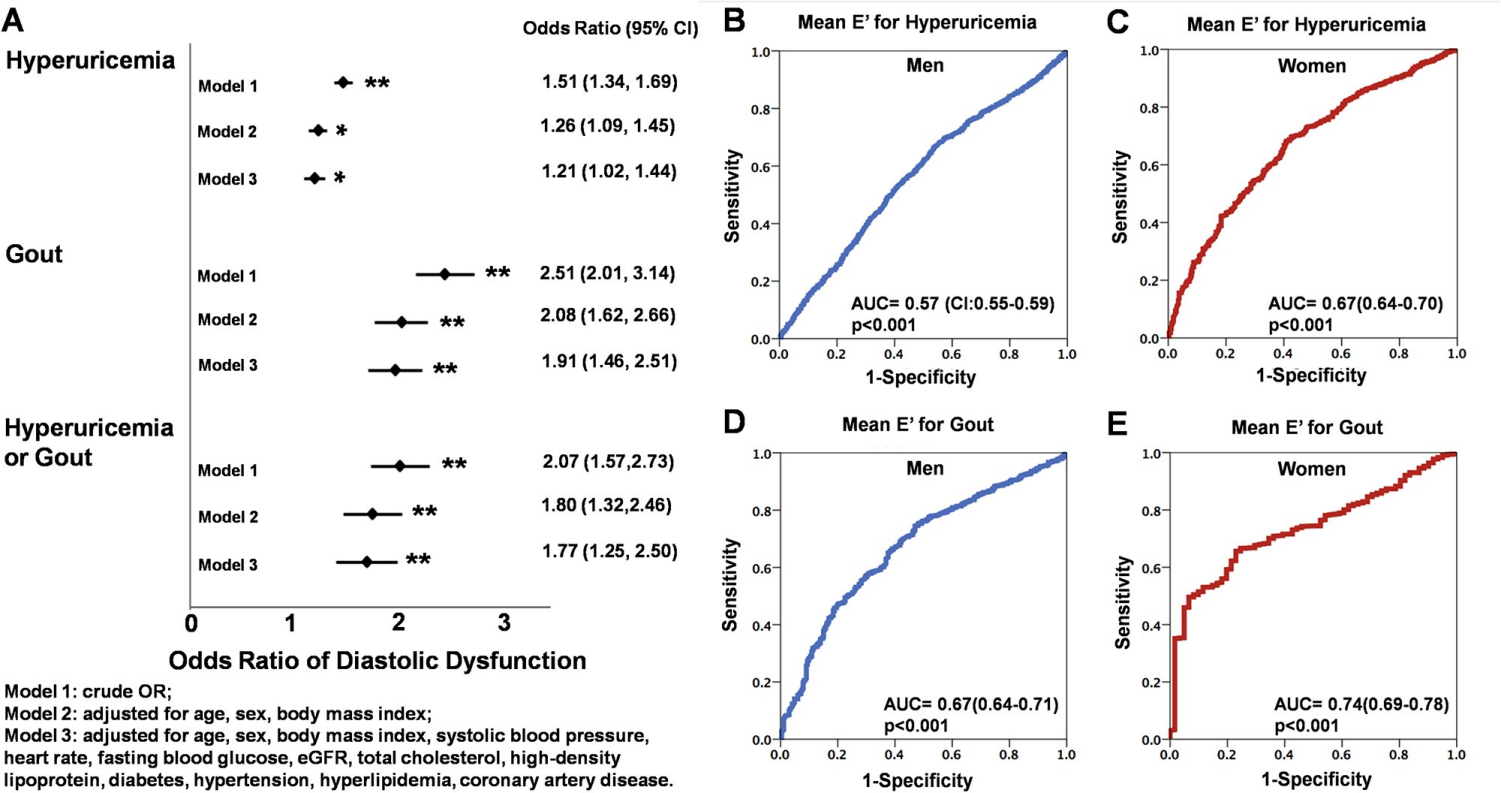
The odds ratio (OR) of hyperuricemia (HU), gout or combined conditions for prevalent diastolic dysfunction (DD). In Fig A, model 1, crude OR; Model 2, adjusted for age, sex, body mass index; Model 3, adjusted for age, sex, body mass index, systolic blood pressure, heart rate, fasting blood glucose, estimated glomerular infiltration rate, total cholesterol, high-density lipoprotein, diabetes, hypertension, hyperlipidemia, and cardiovascular disease. The odds ratio (OR), 95% confidence intervals (95% CI) shown in each group were illustrated with multivariate adjustment. Sex-specific receiver operating characteristic curves (ROC) for LV mitral annular relaxation e’ for men and women for predicting hyperuricemia or gout, with women demonstrating greater area under the ROC than did men (B-E). *denotes p <0.05, ** p <0.001.

Higher SUA levels (<4, 4–6, 6–8, 8–10, >10 mg/dL) (Table 2, all trend p <0.001) and the presence of gout (S1 Table) were both associated with increased LV wall thickness, greater RWT, larger LV volumes, greater LA volumes (with and without index as LAV and LAVi, respectively), and greater LV mass, as well as lower LV e’ and higher LV E/e’. The trends of lower LV e’, higher E/e’ and greater LA volumes (with and without index) remained unchanged by categorizing SUA into quintile groups, especially in women (Fig 1A–1D). Higher SUA was positively associated with greater LV wall thickness (coefficient: 0.03, p = 0.017), RWT (coefficient:0.12, p = 0.044), larger LV mass/volume ratio (coefficient: 0.007, p = 0.037), borderline greater LV mass (indexed) (coefficient: 0.31, p = 0.077), lower mitral annular relaxation e’ (coefficient: -0.086, p <0.001) higher LV E/e’ (coefficient: 0.075, p = 0.019) ad larger LAVi after adjusting for relevant clinical co-variates including age, sex, body mass index, systolic blood pressure, heart rate, fasting blood glucose, estimated glomerular infiltration rate, low-density lipoprotein, high-density lipoprotein, diabetes, hypertension, hyperlipidemia treatment, and cardiovascular disease (Table 2 and Fig 2A–2D). Subjects in the Gout group were similarly associated with more reversed E/A, lower LV e’, higher LV E/e’ and larger LAVi in the multivariate models (S1 Table, all p <0.05). SUA set at 7.0 mg/dL provided the most optimal cut-off for identifying DD according to receiver operating characteristic curves (ROC). The associations among higher SUA, lower e’ (-0.08, [-0.14, -0.02]), higher LV E/e’ (0.11, [0.04, 0.18]) and greater LA volume (1.37, [1.13, 1.61] for LAV; 0.12, [0.001, 0.23] for LAVi, all p<0.05) remained independent in a study subset (n = 3912, 37.2% women) free from histories of hypertension, diabetes, cardiovascular diseases, gout, or evidence of slight to moderate renal functional impairment (defined by eGFR < = 60 mL/min/1.73 m^2^) in both sexes (Fig 1E–1H). Subjects in HU and Gout groups were more likely to demonstrate greater degrees of LV remodeling (S1 Fig: *X*^*2*^: 41.87 and 121.5, respectively, both p <0.001), greater LV mass (with and without index), greater wall thickness, greater RWT, larger LA size (both LAV and LAVi)/LV volumes, lower E/A ratio, and reduced mitral annular relaxation e’ (9.48 vs 8.61 vs 7.46 cm/s) and higher LV E/e’(7.97 vs 8.41 vs 9.44 for Non-HU, HU and Gout, respectively) (Table 3, all p <0.001). A significantly higher risk of diastolic dysfunction (DD) was observed (Non-HU as reference group, OR: 1.51 [1.34–1.69] for HU, OR: 2.51 [2.01–3.14] for Gout, both p <0.001]), and remained significant in multivariate models (adjusted OR for HU: 1.21, p = 0.013; adjusted OR for Gout: 1.91, and adjusted OR for combined HU and Gout group: 1.77, both p <0.001) (Fig 3A).

**Table 3:** Echocardiographic measurement of cardiac structure and diastolic function in non-hyperuricemia, hyperuricemia, and those with gout.

Number, n	Non-adjusted				Multivariate adjusted estimates			
	Non-HU (n = 3610)	HU (n = 1568)	Gout (n = 347)	*p ANOVA*	Non-HU (n = 3610)	HU (n = 1568)	Gout (n = 347)	*p ANOVA*
	n = 3610	n = 1568	n = 347		n = 3610	n = 1568	n = 347	
IVS, mm	8.93±1.13	9.36±1.13*	9.52±1.29*^#^	<0.001	8.92±0.57	9.27±0.49*	9.41±0.53*^#^	<0.001
LVEDV, mL	99.29±18.37	108.52±17.32*	106.46±16.98*	<0.001	100.25±9.7	105.47±9.17*	106.46±8.83*	<0.001
LVESV, mL	32.70±8.40	36.28±9.44*	35.16±7.89*	<0.001	32.86±3.77	34.93±3.43*	35.40±3.48*	<0.001
LVEF, %	67.18±4.70	66.73±5.09*	67.04±4.89	0.046	67.4±0.72	67.0±0.63*	66.9±0.74*^#^	<0.05
LV mass, gm	139.42±32.65	151.37±34.36*	157.07±31.23*^#^	<0.001	139.6±20.56	151.8±18.0*	155.91±19.26*^#^	<0.001
LV mass/volume, gm/ml	1.87±0.28	1.95±0.30*	1.98±0.32*	<0.001	1.86±0.11	1.92±0.10*	1.96±0.11*^#^	<0.001
Relative wall thickness, %	38.7±4.6	39.6±5.0*	39.8±5.0*	<0.001	38.6±1.2	39.3±1.2*	39.7±1.2*^#^	<0.001
LVMi, gm/m^2^ ¥	75.4±15.0	78.1±16.9*	79.8±14.0*	<0.001	75.1±6.4	77.5±5.9*	79.6±6.0*^#^	<0.001
Peak E wave, cm/s	70.32±16.61	66.08±15.45*	65.76±15.36*	<0.001	70.15±5.51	67.50±4.26*	66.87±4.57*	<0.001
Peak A wave, cm/s	60.88±19.55	60.57±18.68	65.40±18.68*^#^	<0.001	60.58±10.57	62.53±11.96*	64.78±9.94*^#^	<0.001
E/A ratio	1.25±0.45	1.17±0.39*	1.08±0.37*^#^	<0.001	1.25±0.25	1.16±0.26*	1.11±0.22*^#^	<0.001
Deceleration time, ms	204.51±38.89	203.50±41.58	207.11±39.98	0.381	203.46±9.86	204.97±12.12*	208.10±10.42*^#^	<0.001
IVRT, ms	89.02±14.85	90.36±15.94	92.83±18.42*	<0.001	89.61±5.08	90.93±5.61*	91.98±4.88*^#^	<0.001
LV e’ (mean), sec	9.48±2.45	8.61±2.30*	7.46±1.98*^#^	<0.001	9.32±1.68	8.77±1.71*	8.37±1.45*^#^	<0.001
LV E/e’ (mean)	7.97±2.56	8.41±2.78*	9.44±2.75*^#^	<0.001	8.07±1.38	8.27±1.55*	8.60±1.31*^#^	<0.001
Maximal LAV, ml	29.7±11.1	33.2±12.9*	34.5±13.1*	<0.001	29.8±5.7	33.2±5.7*	34.2±6.0*^#^	<0.001
Maximal LAVi, ml/m^2^ ¥	16.2±5.7	17.4±6.7*	17.5±6.2*	<0.001	16.1±2.1	16.9±2.2*	17.3±2.1*^#^	<0.001

^¥^ BMI was not included in multi-variate models; Models adjusted for age, sex, body mass index, systolic blood pressure, heart rate, fasting blood glucose, estimated glomerular infiltration rate, low-density lipoprotein, high-density lipoprotein, diabetes, hypertension, hyperlipidemia treatment, cardiovascular disease.

Abbreviations as Table 2.

* p<0.05 vs Non-HU group,

^#^ p<0.05 vs HU group.

### Sex differences on cardiac geometry and diastolic dysfunction in hyperuricemia and gout

While women tended to have lower SUA and had lesser degrees of cardiac remodeling, they demonstrated significantly lower mitral annular relaxation velocity e’ (HU: 8.94 ± 2.29 vs 8.07 ± 2.33 cm/sec; Gout: 7.94 ± 1.98 vs 7.26 ± 1.73 cm/sec, for men and women) and markedly higher LV E/e’ (HU: 7.84 ± 2.37 vs 9.79 ± 3.39; Gout: 8.69 ± 2.50 vs 9.93 ± 2.38, for men and women, respectively, all p<0.05) in both HU and Gout groups; there were no differences on LV e’ by gender in the Non-HU group (S2 Fig). There was markedly larger LAVi (16.4 ± 5.9 vs 19.7 ± 7.9 for men and women, p<0.001) in women with clinical HU but not in Gout group compared to their male counterparts; however, in general, women showed greater LV E/e’(7.60±2.28 vs 9.54±3.32, p<0.001 for men and women) and larger LA size (LAVi: 17.4±6.6 vs 16.1±5.7 ml for women and men, respectively, p<0.001) than did men. Notably, no differences on medications use for HTN and gouty arthritis were observed between sex (S2 Table). Overall, there was a larger area under the ROC curve (AUROC) for LV e’ for predicting Gout (0.74, [95% CI: 0.69–0.78] vs 0.67, [95% CI: 0.64–0.70] for women vs men, p for ΔAUROC: 0.035) and HU (0.67, [95% CI: 0.64–0.70] vs 0.57, [95% CI: 0.55–0.59] for women vs men, p for ΔAUROC: <0.001) (Fig 3B–3E). The optimal LV e’ cut-off set at 9.12 cm/sec in women and e’ set at 8.26 cm/sec in men identified the Gout group.

## Discussion

Our study is the largest to date to demonstrate the relationships among SUA levels, diastolic function, and LV remodeling in an asymptomatic ethnic Asian population. There were three main findings: (1) Both hyperuricemia and gout groups had greater LV mass indexes, more concentric remodeling, and worse LV diastolic function that was more pronounced in the gout group; (2) Elevated SUA levels were related to concentric LV, ventricular hypertrophy, and worse diastolic function; and (3) In individuals with hyperuricemia, women demonstrated greater degrees of concentric LV remodeling and worse diastolic function than did men.

### The influence of hyperuricemia or gout on systemic inflammation and LV remodeling

Increased production of uric acid promotes LV hypertrophy and cardiac fibrosis by stimulating inflammation and oxidative stress, thereby leading to diastolic dysfunction [15]. Uric acid is the end-product of purine metabolism, catalyzed by xanthine oxidase (XO). It is believed to play an important role in chronic inflammation. Hyperuricemia results from increased production or decreased excretion of serum urate. Gout occurs when monosodium urate crystals deposit in the joints or soft tissues, causing acute arthritis. Both chronic hyperuricemia and gout involve inflammation responses and stimulate the release of interleukin-1β, interleukin-6 (IL-6), tumor necrosis factor-α (TNF- α), NF-kβ, and activate the NALP3 inflammasome complex [16–18]. Pro-inflammatory cytokines induce LV hypertrophy and contribute to ventricular impairment [15, 19, 20]. Beyond the effect that uric acid may have as an inflammatory mediator in the pathogenesis of LV remodeling and diastolic dysfunction, several lines of evidence suggest that up-regulation of XO occurs in left ventricular hypertrophy and heart failure in both human and animal models [21, 22].

### The influence of hyperuricemia and gout on LV diastolic function

Increased XO pathway activity produces free superoxide radicals and consequent heart failure. Inhibition of XO activity causes left ventricular regression and LV contractile functional improvement by way of SUA reduction [23, 24]. Interestingly, in the present study, individuals with gout had worse diastolic function than did the normal and hyperuricemia groups. In clinical practice, patients with gouty arthritis receive more aggressive therapy for hyperuricemia and hence have lower SUA levels than do asymptomatic individuals with hyperuricemia. Despite the occurrence or repetitive episodes of acute inflammation in gout attacks, lower SUA levels may contribute to less LV hypertrophy in the gout group than in the hyperuricemia group. In terms of inflammatory status, reactive oxidase species may limit nitric oxide bioavailability to the myocardium, thereby stimulating cardiomyocyte stiffness beyond pathways of cardiac hypertrophy in the pathogenesis of heart failure with preserved ejection fraction (HFpEF) [25]. Through activating inflammatory responses and the RAS, chronic hyperuricemia and gout may lead to cardiac fibrosis, LV hypertrophy, and passive ventricular stiffness that cause diastolic dysfunction. While current Japanese guideline for management of hyperuricemia and gout strongly recommended pharmacological intervention for subjects with SUA levels greater than 9.0 mg/dL or those reaching 8.0 mg/dL with clinical complications (including renal damage, urinary lithiasis, hypertension, and diabetes), we found that SUA levels around 7.0 mg/dL were accompanied by some level of cardiac dysfunction in our asymptomatic Asian population [25].

### Sex differences in hyperuricemia and gout-related diastolic function

Women with hyperuricemia appeared to be more susceptible to the development of diastolic dysfunction than did men, even after adjustment for diuretics use [26]. To date, the sex-related differences in cardiac remodeling and diastolic impairment in individuals with chronic hyperuricemia and gout remain unclear. On the one hand, we observed that both LV remodeling in terms of LV mass index and LV mass/volume ratio were equal or tended to be lower in women than men in HU or Gout groups; on the other hand, consistent with results of a prior study, women in general may experience greater degrees of diastolic dysfunction (lower e’, or greater E/e’) than do men [27]. Taken together, these data suggest that unfavorable LV concentricity and remodeling may not be contributing factors for the development of diastolic functional impairment in women with hyperuricemia and gout. Instead, the development of diastolic dysfunction might stem from lower LV compliance or increased intrinsic myocardial stiffness from elicited extracellular collagen deposition beyond LV concentricity and hypertrophy [28]. While higher XO activity was observed in women than men [29], we speculate that larger amounts of free superoxide radicals may be generated in women in the context of metabolic disorders, leading to worsening diastolic parameters, more impaired LV compliance, and susceptibility to the development of HFpEF [26, 30, 31].

The link between hyperuricemia and clinical diastolic dysfunction (DD) (adjusted OR: 1.21 and 1.91, respectively) may start at a relatively low clinical cut-off point (7.0 mg/dL), demonstrating independent associations with impaired myocardial relaxation e’ and ventricular filling E/e'.

While men and women showed similar degree of cardiac remodeling, women in general presented with worse diastolic parameters than did men.

## Limitations

The major limitation of our research is its single center, observational, and retrospective nature; our results may not be representative of the general population. As a cross-sectional study design, our current findings failed to explain the causal relationship. Second, there was a relatively small sample size to determine associations between SUA levels and LV diastolic function; nevertheless, we provided detailed data regarding the relationship between diastolic function and LV remodeling in a large asymptomatic ethnic Asian cohort. Third, the number of females was relatively small in the hyperuricemia and gout groups; therefore the findings regarding sex differences should be interpreted carefully.

## Conclusion

In a large Asian population, hyperuricemia was both tightly linked to unfavorable LV remodeling and was independently associated with impaired diastolic dysfunction, even at relatively low values. These structural and functional alterations were more pronounced in subjects with known gout history. Women in general experienced less pronounced cardiac remodeling with hyperuricemia or gout, despite the fact that they a demonstrated greater degree of diastolic functional impairment than did men, with underlying comparable diastolic function in non-hyperuricemia status. Our data suggest that SUA may serve as a marker for unfavorable cardiac remodeling or pre-clinical heart failure marker at very early stages, especially for women.

## Supporting information

S1 Table Cardiac structure remodeling and diastolic indices in subjects with diagnosis of gout. (DOCX)

S2 Table Comparisons of hypertension and gout medication use in study subjects. (DOCX)

S1 Fig Hyperuricemia and gout group correlate with higher percentage of cardiac remodeling and impaired LV diastolic dysfunction. (DOCX)

S2 Fig Association between serum uric acid level, cardiac structural remodeling and diastolic indices. (DOCX)
