# Supplementary material for: Associations of serum uric acid level and gout with cardiac structure, function and sex differences from large scale asymptomatic Asians
Source: PLoS One. 2020 Jul 20;15(7):e0236173. doi: 10.1371/journal.pone.0236173 (PMC7371161; doi:10.1371/journal.pone.0236173)
Supplement: S1 Table — (DOCX) [file pone.0236173.s001.docx]

**Table S1. Cardiac structure remodeling and diastolic indices in subjects with diagnosis of Gout**

|  | **No Gout: -** | **Gout: +** | **P value (T test)** | **Univariate Linear Regression**  **(Gout: +)** | | **Multivariate Linear Regression**  **(Gout: +)** | |
| --- | --- | --- | --- | --- | --- | --- | --- |
| **Echocardiography Indices** |  |  |  | **Coef: (95% CI)** | **p value** | **Coef: (95% CI)** | **p value** |
| IVS, mm | 9.0±1.1 | 9.4±1.3 | <0.001 | 0.42 | <0.001 | 0.07 | 0.243 |
| LVEDV, ml | 76.1±14.5 | 79.7±13.1 | <0.001 | 3.62 | <0.001 | -0.48 | 0.505 |
| LVESV, ml | 28.6±7.6 | 29.9±7.1 | 0.003 | 1.29 | 0.003 | -0.43 | 0.289 |
| LVEF, % | 62.6±5.5 | 62.6±5.7 | 0.90 | 0.09 | 0.757 | 0.42 | 0.147 |
| LV mass, g/m^2^ | 142.5±33.9 | 156.2±32.9 | <0.001 | 13.7 | <0.001 | 0.52 | 0.739 |
| LV M/V ratio, gm/ml | 1.88±0.31 | 1.97±0.32 | <0.001 | 0.09 | <0.001 | 0.02 | 0.307 |
| LVMi, gm/m^2^ ¥ | 76.2±15.6 | 79.8±14.0 | <0.001 | 3.81 | <0.001 | 0.98 | 0.237 |
| Peak E wave, cm/s | 69.4±16.5 | 65.8±15.4 | <0.001 | -3.67 | <0.001 | -0.63 | 0.531 |
| Peak A wave, cm/s | 60.8±19.4 | 65.4±18.7 | <0.001 | 4.59 | <0.001 | 1.46 | 0.167 |
| E/A ratio | 1.23±0.44 | 1.08±0.37 | <0.001 | -0.16 | <0.001 | -0.05 | 0.028 |
| Deceleration time, ms | 204.3±39.5 | 207.1±40.0 | 0.23 | 2.81 | 0.233 | -0.94 | 0.704 |
| IVRT, msec | 89.3±15.1 | 92.8±18.4 | <0.001 | 3.54 | <0.001 | 0.5 | 0.602 |
| Mean e’, cm/s | 9.24±2.44 | 7.81±1.94 | <0.001 | -1.43 | <0.001 | -0.05 | 0.028 |
| Mean E/e’ | 8.10±2.66 | 8.92±2.52 | <0.001 | 0.82 | <0.001 | 0.35 | 0.018 |
| Maximal LAV, ml | 30.7±11.8 | 34.5±13.1 | <0.001 | 3.83 | <0.001 | 1.11 | 0.07 |
| Maximal LAVi, ml/m^2^ ¥ | 16.5±6.1 | 17.5±6.2 | 0.007 | 1.00 | 0.007 | 0.73 | 0.042 |

**Abbreviations as Table 2.**

¥ BMI was not included in multi-variate models;

Multivariate models were adjusted for age, sex, body mass index, systolic blood pressure, heart rate, fasting blood glucose, estimated glomerular infiltration rate, low-density lipoprotein, high-density lipoprotein, diabetes, hypertension, hyperlipidemia, cardiovascular disease.
