# Supplementary material for: Associations of serum uric acid level and gout with cardiac structure, function and sex differences from large scale asymptomatic Asians
Source: PLoS One. 2020 Jul 20;15(7):e0236173. doi: 10.1371/journal.pone.0236173 (PMC7371161; doi:10.1371/journal.pone.0236173)
Supplement: S2 Table — (DOCX) [file pone.0236173.s002.docx]

**Table S2 Comparisons of hypertension and gout medication use in study subjects**

| **HTN (Total n = 1121)** | **Male (n=722)** | **Female (n=399)** | *p (ꭕ^2^)* |
| --- | --- | --- | --- |
| ACEi/ARB, number (%) | 80 (11.1%) | 32 (8.0%) | 0.12 |
| CCB, number (%) | 145 (20.1%) | 71 (17.8%) | 0.35 |
| BB, number (%) | 124 (17.2%) | 69 (17.3%) | 0.96 |
| Diuretics, number (%) | 174 (24.1%) | 98 (24.6%) | 0.86 |
| **Gout (Total n = 347)** | **Male (n=285)** | **Female (n=62)** | *p (ꭕ^2^)* |
| Medications for Gout | 94 (33.0%) | 20 (32.3%) | 0.91 |

ACEi: angiotensin- converting enzyme inhibitors; ARB: angiotensin receptor blockers; BB: β-blocker; CCB: calcium channel blocker;
