# Supplementary material for: Associations of serum uric acid level and gout with cardiac structure, function and sex differences from large scale asymptomatic Asians
Source: PLoS One. 2020 Jul 20;15(7):e0236173. doi: 10.1371/journal.pone.0236173 (PMC7371161; doi:10.1371/journal.pone.0236173)
Supplement: S1 Fig — (DOCX) [file pone.0236173.s003.docx]

**Fig S1 Hyperuricemia and Gout group correlate with higher percentage of cardiac remodeling and impaired LV diastolic dysfunction**

**
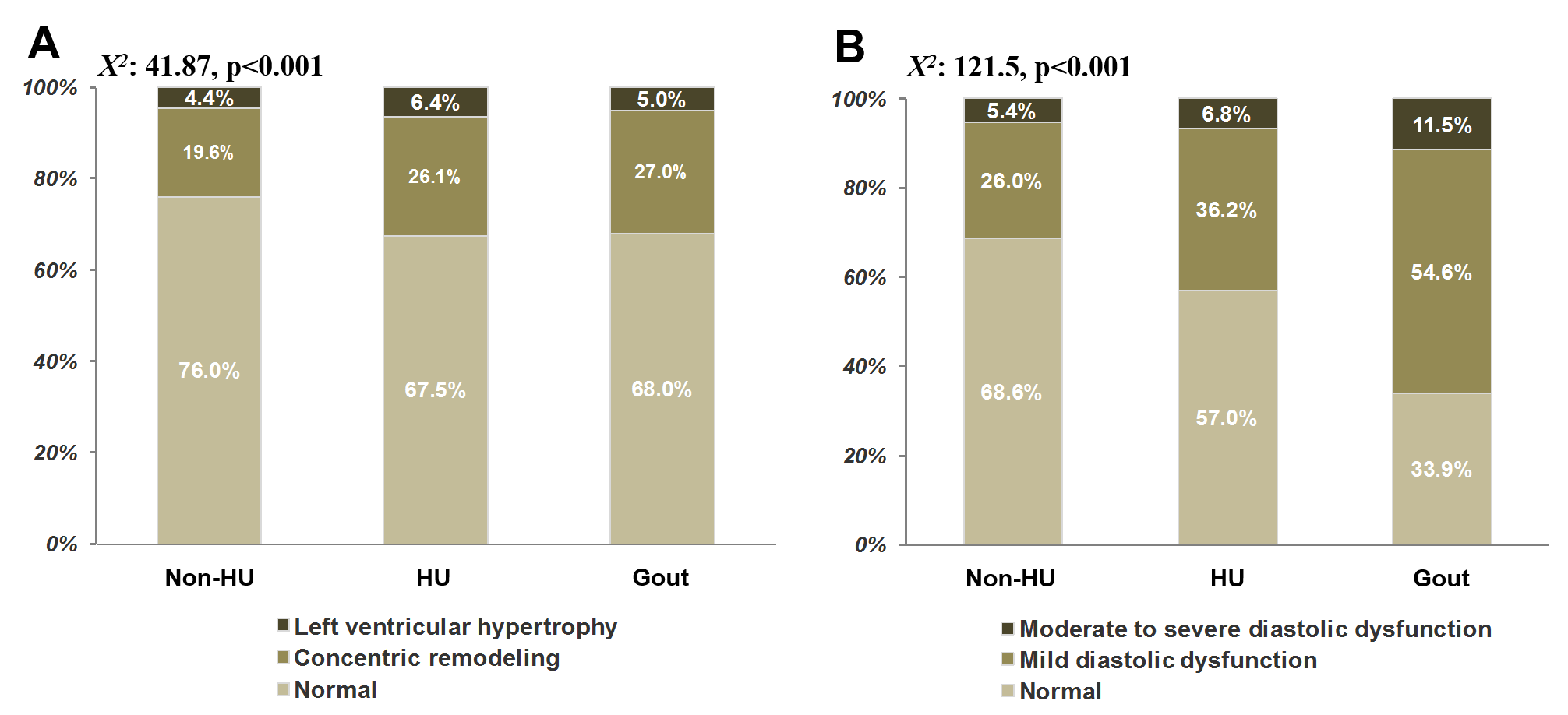
**
