# Supplementary material for: Associations of serum uric acid level and gout with cardiac structure, function and sex differences from large scale asymptomatic Asians
Source: PLoS One. 2020 Jul 20;15(7):e0236173. doi: 10.1371/journal.pone.0236173 (PMC7371161; doi:10.1371/journal.pone.0236173)
Supplement: S2 Fig — (DOCX) [file pone.0236173.s004.docx]

**Fig S2. Association between serum uric acid level, cardiac structural remodeling and diastolic indices**

**
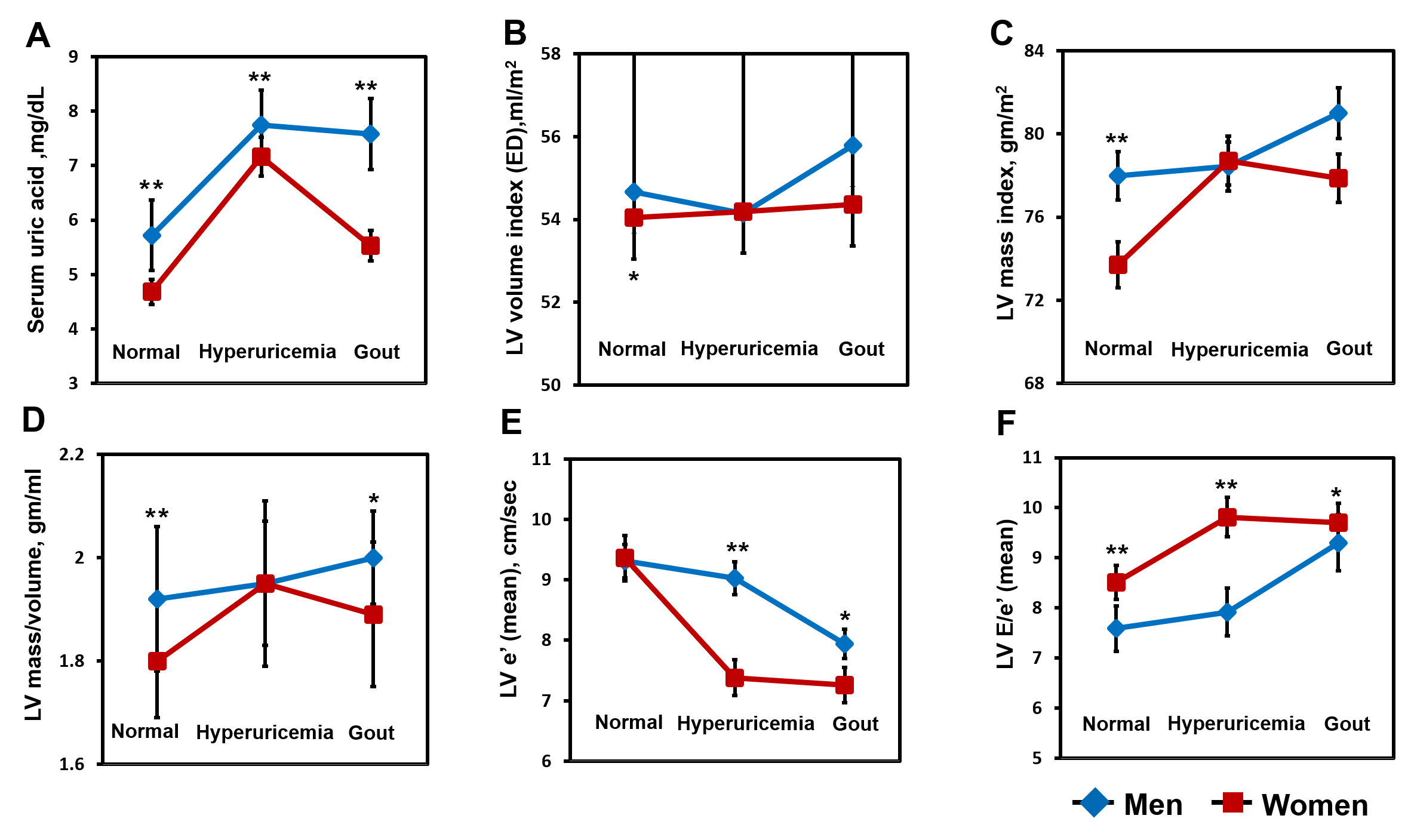
**

*: p<0.05, **: p<0.001 comparison between men and women. Error bars indicate standard deviation (SD) in each group.

ED: end-diastolic phase; HU: hyperuricemia; LV: left ventricular; LV e’ (mean): averaged TDI-determined lateral and septal mitral annular relaxation velocity e’; LV E/e’ (mean): Mitral inflow E to averaged mitral annular relaxation velocity e’.
